# GCSA-SegFormer: Transformer-Based Segmentation for Liver Tumor Pathological Images

**DOI:** 10.3390/bioengineering12060611

**Published:** 2025-06-04

**Authors:** Jingbin Wen, Sihua Yang, Weiqi Li, Shuqun Cheng

**Affiliations:** 1 School of Biomedical Engineering, Southern Medical University, No. 1023-1063, Shatai South Road, Baiyun District, Guangzhou 510440, China; 2 School of Health Science and Engineering, University of Shanghai for Science and Technology, Shanghai 200082, China; 3 Department of Hepatic Surgery VI, Eastern Hepatobiliary Surgery Hospital, Second Military Medical University, Shanghai 200082, China

**Keywords:** pathological images, deep learning, global channel spatial attention, GCSA-SegFormer

## Abstract

Pathological images are crucial for tumor diagnosis; however, due to their extremely high resolution, pathologists often spend considerable time and effort analyzing them. Moreover, diagnostic outcomes can be significantly influenced by subjective judgment. With the rapid advancement of artificial intelligence technologies, deep learning models offer new possibilities for pathological image diagnostics, enabling pathologists to diagnose more quickly, accurately, and reliably, thereby improving work efficiency. This paper proposes a novel Global Channel Spatial Attention (GCSA) module aimed at enhancing the representational capability of input feature maps. The module combines channel attention, channel shuffling, and spatial attention to capture global dependencies within feature maps. By integrating the GCSA module into the SegFormer architecture, the network, named GCSA-SegFormer, can more accurately capture global information and detailed features in complex scenarios. The proposed network was evaluated on a liver dataset and the publicly available ICIAR 2018 BACH dataset. On the liver dataset, the GCSA-SegFormer achieved a 1.12% increase in MIoU and a 1.15% increase in MPA compared to baseline models. On the BACH dataset, it improved MIoU by 1.26% and MPA by 0.39% compared to baseline models. Additionally, the performance metrics of this network were compared with seven different types of semantic segmentation, showing good results in all comparisons.

## 1. Introduction

The liver is not only the largest gland in the human body but also the largest solid organ. Primary liver cancer ranks sixth among malignant tumors, with more than half of new cases occurring in China each year. The prognosis for liver cancer patients is generally poor, with a high mortality rate. Primary liver cancer includes hepatocellular carcinoma (HCC), intrahepatic cholangiocarcinoma, and liver sarcoma, with HCC being the fifth most common malignant tumor in China, accounting for approximately 400,000 new cases annually. Its mortality rate is second only to lung cancer. Surgery is the most commonly used curative treatment for liver cancer; however, the 5-year recurrence rate after resection is as high as 70%, and even among patients who meet the Milan criteria for liver transplantation, the 5-year recurrence rate remains at 20%. Microvascular invasion (MVI), defined as the presence of cancer cell nests within the lumens of endothelial-lined blood vessels observed microscopically [1], is one of the critical factors affecting postoperative prognosis [2,3,4].

Clinical doctors can initially assess the liver cell lesion area roughly with the help of radiological images, but for more precise diagnosis, pathologists need to further analyze the pathological images [5]. Pathological images, also known as Whole Slide Imaging (WSI), utilize high-resolution slicing scanning techniques to scan pathological tissues obtained from surgery or biopsy, providing detailed tissue structure information that serves as crucial evidence for disease diagnosis and treatment. Pathological images are considered the “gold standard” for tumor diagnosis and play an irreplaceable role in ensuring diagnostic accuracy and treatment effectiveness [6]. The pixel count of liver pathological images can range from millions to billions. Due to current GPU memory limitations, directly inputting WSI images with millions to billions of pixels into popular deep learning models is impractical [7]. Segmentation of WSI images to obtain reduced images or small image blocks is commonly used as input data. However, downsampling can lead to the loss of critical features. Therefore, the WSI was segmented into patches to serve as inputs for the network.

Artificial intelligence (AI) is an advanced technology involved in nearly every discipline today. It can rapidly screen for various lesions in pathological images, assisting doctors in making swift and accurate diagnoses while helping experts analyze details and features that are difficult to discern with the naked eye [8].

In the field of computer vision, the development of convolutional neural networks (CNNs) has significantly advanced the technology. Since the breakthrough performance of AlexNet in the ImageNet competition in 2012, CNNs have become the mainstream architecture for tasks such as image classification, object detection, and semantic segmentation. The introduction of Transformers [9] in 2017 by Vaswani et al. provided a new perspective on visual tasks, leveraging self-attention mechanisms that initially excelled in natural language processing (NLP) and gradually transitioned to computer vision. SegFormer [10], as an innovative representative in this domain, combines the strengths of Transformers and CNNs, achieving outstanding performance in semantic segmentation tasks. SegFormer not only enhances segmentation accuracy but also significantly reduces computational costs.

In this paper, we propose a novel Global Channel Spatial Attention (GCSA) module designed to enhance the representational capability of input feature maps. This module integrates channel attention, channel shuffling, and spatial attention, effectively capturing global dependencies within feature maps. By incorporating the GCSA module into the SegFormer architecture, the resulting network, named GCSA-SegFormer, can more accurately capture global information and detailed features in complex scenarios. We validated the model’s effectiveness using authoritative pathological image datasets provided by Eastern Hepatobiliary Hospital and the publicly available ICIAR 2018 BACH dataset. Our model was compared against the original SegFormer and seven other semantic segmentation models.

## 2. Background and Related Work

Semantic segmentation is a computer vision technique that aims to partition an image into different regions with semantic information, assigning category labels to each pixel such as people, cars, trees, etc., to achieve detailed image segmentation. In the field of medical imaging, semantic segmentation plays a crucial role, enabling precise localization and segmentation of lesion areas, identification of organ structures, assisting doctors in disease diagnosis, treatment planning, and surgical navigation, thereby improving diagnostic accuracy and treatment effectiveness.

The pioneering work in semantic segmentation is FCN [11], which first achieved end-to-end pixel-level segmentation; the U-Net [12] proposed in the same year performed excellently in medical image segmentation with its U-shaped architecture. Subsequent improved models, such as Attention U-Net, Residual U-Net, etc., further enhanced performance. In 2017, PSPNet [13] introduced the pyramid pooling module to capture multi-scale contextual information. The DeepLab series (such as DeepLabV3 [14], DeepLabV3+ [15]) improved segmentation accuracy through dilated convolutions and ASPP modules. Additionally, LR-ASPP [16] reduced computational complexity and improved efficiency by using low-resolution dilated convolutions. HRNet [17], proposed in 2019, captured multi-scale information through high-resolution feature propagation. In recent years, models based on Transformers have shown advantages in handling global context and long-range dependency relationships suitable for complex pathological image segmentation.

In the field of liver tumor pathological image segmentation, researchers have proposed various innovative methods. Zhou et al. [18] introduced a framework based on support vector machines, initializing segmentation with classifiers and optimizing results using auxiliary tools. Christ et al. [19] employed cascaded FCNs and dense 3D conditional random fields (CRFs) to achieve automatic segmentation of liver and lesion areas in abdominal CT images. Chen et al. [20] proposed a two-step segmentation method based on fractal residual FRA-UNet, refining tumor segmentation results with fully connected CRFs. Xu et al. [21] improved the segmentation accuracy of colorectal cancer liver-cell metastasis pathological images by using multi-scale features and deep convolutional neural networks. Zhai et al. [22] introduced an attention-guided connection module, combined with pyramid sampling operations, further enhancing the segmentation performance of liver cancer pathological images. The H-DenseUNet model proposed by Li et al. [23] segments the liver and tumors from CT volumes, while Han et al. [24] combined the architectures of U-Net and ResNet to achieve a similar task. Tian et al. [25] proposed a multimodal framework that combines segmentation models and language models to extract information from CT slices and text reports. Abdalla et al. [26] used a hybrid network of 2D and 3D DenseU-Nets to segment the liver and tumors in CT images. Zheng et al. [27] refined segmentation results using a 3D deformable model based on an FCN model. Wang [28], Chlebus [29], and others used a three-plane FCN network for liver and tumor segmentation. Pan et al. [30] improved the classification accuracy of liver cell carcinoma by combining a 3D-CNN model with temporal features from ultrasound videos. Schmauch et al. [31] used a CNN model based on ResNet50 to simultaneously handle malignant tumor detection and lesion type characterization tasks.In addition, a deep learning method [32] combining a spatial attention mechanism and background removal algorithm has been proposed for efficient analysis of tumor cells in WSI images, demonstrating a strong capability for pathology image assessment.

Although significant progress has been made in liver tumor segmentation on CT images, research on pathological image segmentation is relatively limited. Pathological images have higher resolution and complexity, posing greater challenges for segmentation tasks. Transformer-based segmentation models are suitable for handling such medical images and are expected to bring some advancements in the field of pathological image segmentation.

## 3. Datasets and Method

### 3.1. Datasets

To evaluate the performance of the proposed network, the dataset consists of pathological images of liver cancer stained with Hematoxylin–Eosin (HE) from 71 cases provided by the Department of Pathology at the Eastern Hepatobiliary Hospital of the Chinese People’s Liberation Army Naval Medical University, along with their corresponding annotations. This dataset will be referred to as the liver dataset. The annotations include regions of interest (ROIs), HCC, MVI, and regions of non-interest (Non-ROIs). It is worth noting that there may be some regions with incomplete annotations in the dataset. For the purposes of this study, the MVI and HCC areas are combined and collectively referred to as the foreground, with pixel values set to 255. The ROI and Non-ROI areas are combined and referred to as the background, with pixel values set to 0.

**Figure 1:**
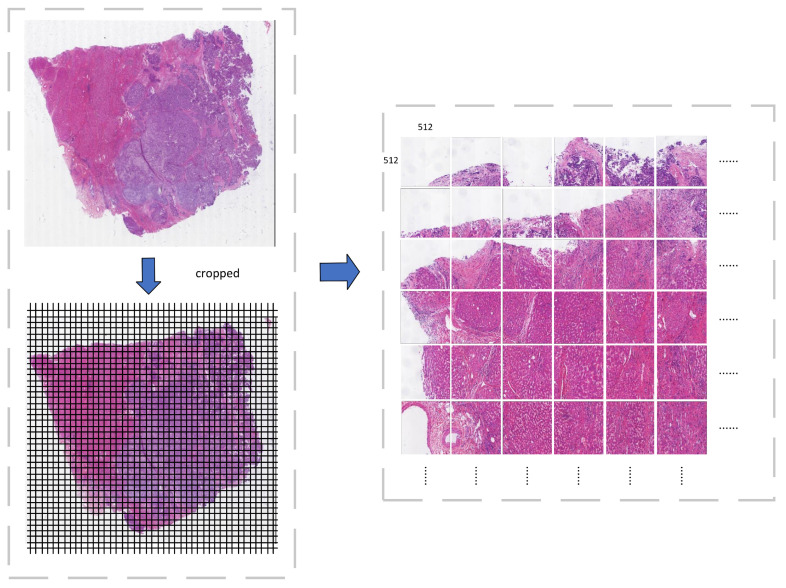
Image preprocessing: crop the Whole Slide Image into patches of size 512 × 512 pixels.

Due to current GPU memory limitations, it is impractical to directly input millions to billions of pixels from WSI into deep learning models. Additionally, considering that the final predicted patches will be compiled into a complete WSI, image preprocessing is required before inputting them into the network (Figure 1). The 71 WSI images were processed using the Pillow (PIL) library for fixed-size cropping, dividing each image into 512 × 512 patches. A total of 56,833 patches were generated and partitioned into training, validation, and testing sets with a ratio of 7:2:1.

We also selected the publicly available ICIAR 2018 BACH Task 2 dataset, which contains 10 labeled WSI images and 20 unlabeled WSI images, hereafter referred to as the BACH dataset. In this dataset, normal tissue and benign lesion areas are combined into the background, with pixel values set to 0. The in situ carcinoma and invasive carcinoma regions are combined into the foreground, with pixel values set to 255. By cutting the labeled WSI images horizontally, we generated a total of 10,342 image patches, each measuring 512 × 512 pixels. Finally, these image patches were also divided into training, validation, and testing sets with a ratio of 7:2:1.

### 3.2. Method

**Figure 2:**
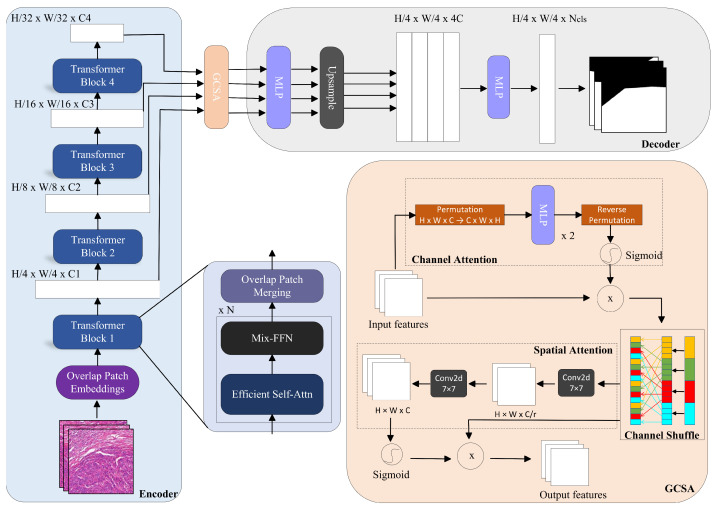
GCSA-SegFormer based on the SegFormer architecture: global channel spatial attention is introduced at the connection between the encoder and decoder.

Our method is based on the SegFormer architecture, incorporating a global channel spatial attention at the connection between the encoder and decoder (Figure 2).

The SegFormer encoder utilizes multiple Transformer layers, each containing multi-head attention mechanisms and feedforward neural networks. These layers extract features and perform representation learning from pathological images. The multi-head attention mechanism helps the model capture dependencies between tissue structures and lesion areas, while the feedforward neural network applies nonlinear transformations and integration to enhance the depiction and analysis of complex pathological features.

The GCSA module in Figure 2 consists of three components: channel attention, channel shuffling, and spatial attention, which further process the features output by the encoder.

(1) Channel Attention

In the processed liver pathological images from the SegFormer encoder, inter-channel relationships may be overlooked, yet these relationships are crucial for capturing important global information in the images. Without considering inter-channel dependencies, the model may fail to fully utilize all information in the feature maps, resulting in insufficient capture of lesion characteristics.

To enhance the inter-channel dependencies, we employ a multi-layer perceptron (MLP) in the channel attention module. This approach reduces redundancy among channels and emphasizes important features. To facilitate this, we perform a dimensional permutation on the feature maps output by the SegFormer encoder—specifically, we rearrange the tensor such that the channel vectors at each spatial location can be treated as individual feature vectors for the MLP. This enables the MLP to learn relationships across channels at each pixel position while preserving spatial information. The channel vectors are then passed through two fully connected layers. The first layer reduces the number of channels to one-fourth of the original, introducing nonlinearity via a ReLU activation function. The second layer restores the channel count to the original dimensions. This process effectively models local dependencies between channels. Finally, we reverse the permutation to restore the original shape and apply a Sigmoid activation function to generate the channel attention map. The attention map is then element-wise multiplied with the original input feature map to produce the enhanced output.

(1) $$\begin{array}{cc} F_{C} & =\sigma \left(\mathrm{MLP}\left(\mathrm{Permute}\left(F_{\mathrm{IN}}\right)\right)\right)\cdot F_{\mathrm{IN}.} \end{array}$$

where $F_{C}$ represents the enhanced feature map, σ denotes the Sigmoid function, · indicates multiplication, and $F_{\mathrm{IN}}$ refers to the input feature map.

(2) Channel Shuffling

After enhancing the liver pathological images with the channel attention, there may still be cases where inter-channel information is not adequately mixed. Insufficient mixing of channel information can limit the expressive capability of the features, preventing the model from fully leveraging the advantages of channel attention.

To further mix and share information, we implement a channel shuffling operation. The feature map enhanced by the channel attention mechanism is divided into four groups, each containing $\frac{C}{4}$ channels. We then perform a transposition operation on the grouped feature maps to disrupt the order of the channels within each group. Finally, the shuffled feature maps are restored to their original shape. This approach allows for better mixing of feature information, thereby enhancing the feature representation capability.

(2) $$\begin{array}{c} F_{CS}=\mathrm{ChannelShuffle}\left(F_{C}\right). \end{array}$$

where $F_{CS}$ represents the feature map after channel shuffling, and $F_{C}$ refers to the input feature map.

(3) Spatial Attention

After the channel attention and channel shuffling, while inter-channel information has received further attention in processing liver pathological images, there may still be cases where spatial information is not fully utilized. Spatial information is crucial for capturing both local and global features within an image. Ignoring the spatial dimensions can lead the model to miss critical details in the image.

To address this issue, we employ a spatial attention that processes spatial information through two 7×7 convolutional layers. First, the input feature map is passed through a 7×7 convolutional layer, reducing the number of channels to one-fourth of the original. This is followed by batch normalization and a ReLU activation function for nonlinear transformation. Next, the feature map is processed through a second 7×7 convolutional layer, restoring the channel count to the original dimensions, followed by another batch normalization layer. This approach effectively captures the dependencies in the spatial dimensions. Finally, we generate the spatial attention map using a Sigmoid activation function. The shuffled feature map is then element-wise multiplied by the spatial attention map to produce the final output feature map.

(3) $$\begin{array}{c} F_{S}=\sigma \left({\mathrm{Conv}}^{7\times 7}\left(\mathrm{BN}\left(\mathrm{ReLU}\left({\mathrm{Conv}}^{7\times 7}\left(F_{CS}\right)\right)\right)\right)\right)\cdot F_{CS}. \end{array}$$

where $F_{S}$ represents the feature map after applying spatial attention, · indicates multiplication, and $F_{CS}$ refers to the input feature map.

The SegFormer decoder employs multi-layer feature aggregation and a lightweight MLP design. It performs upsampling and reconstruction on the features processed by the GCSA module, integrating multi-scale features to generate a feature map of size $\frac{H}{4}$, $\frac{W}{4}$, 4C. This feature map has one-quarter of the original height and width, with the number of channels increased by a factor of four. Finally, an MLP is used for the final classification, outputting the corresponding number of classes.

## 4. Experiments and Results

### 4.1. Experimental Environment and Parameters

The experimental environment is based on Python 3.8, PyTorch 1.8.1, and CUDA 11.1. The operating system is Ubuntu 18.04, and the GPU used is a 24GB NVIDIA RTX 3090. The backbone feature extraction network for all models is pre-trained on the ImageNet dataset. We choose the trunk feature extractor that is pre-trained on Imagenet, mainly based on the current mainstream visual Transformer structure, which generally adopts Imagenet as the pre-training standard. During the training process, the AdamW optimizer is employed to optimize the network parameters, with a momentum set to 0.9, weight decay set to 0.02, and a learning rate of 0.0001. The learning rate decay method is set to step, with a total of 50 epochs and batch size of eight. To ensure fairness and reproducibility across models, all experiments were conducted using the same dataset splits and a fixed random seed. Therefore, the reported results correspond to a single run per model under controlled settings.

### 4.2. Evaluation Metrics

To quantitatively assess the segmentation performance of different network models on pathological images, this study employs three evaluation metrics: Mean Intersection over Union (MIoU), Mean Pixel Accuracy (MPA), and accuracy. MIoU calculates the intersection over union ratio between predicted results and ground truth labels. MPA measures the model’s pixel-level classification accuracy in image segmentation tasks. Accuracy can be interpreted as a measure of the model’s accuracy in classifying each pixel. The values of these three metrics are in the range [0, 1]. All three metrics approach a value of 1, indicating better segmentation performance. The calculations for these metrics are as follows:

(4) $$\begin{array}{c} \mathrm{MIoU}=\frac{\left(\frac{TP}{TP\;+\;FP\;+\;FN}+\frac{TN}{TN\;+\;FN\;+\;FP}\right)}{2}, \end{array}$$

(5) $$\begin{array}{c} \mathrm{MPA}=\frac{\left(\frac{TP}{TP\;+\;FP}+\frac{TN}{TN\;+\;FN}\right)}{2}, \end{array}$$

(6) $$\begin{array}{c} \mathrm{Accuracy}=\frac{TP\;+\;TN}{TP\;+\;FN\;+\;FP\;+\;TN}. \end{array}$$

where true positive (*TP*) refers to the correctly predicted foreground pixel positions, false positive (*FP*) refers to the background pixel positions incorrectly predicted as foreground, false negative (*FN*) refers to the foreground pixel positions incorrectly predicted as background, and true negative (*TN*) refers to the correctly predicted background pixel positions.

### 4.3. Results and Analysis

#### 4.3.1. Liver Dataset

In pathological image analysis, the choice and design of the model are crucial for segmentation performance. Different network architectures have their own advantages, adapting to various application needs and data characteristics. To gain deeper insights into the performance of these models in practical applications, we conducted a detailed analysis of the performance of several mainstream segmentation models based on the experimental results. The effectiveness of these models reflects not only their ability to capture details and extract features but also reveals their limitations in handling complex structures and multi-scale information.

From the experimental results on the liver dataset presented in Table 1, we observe significant performance differences among the various models in pathological image segmentation. The DeepLabv3+ model, which employs dilated convolutions to expand the receptive field, is well suited for capturing complex pathological structures. However, its use of the lightweight MobileNetV2 as the backbone network may hinder its performance in large-scale feature aggregation. In contrast, DeepLabv3 shows slightly inferior performance when dealing with edges and complex structures in pathological images. Although the pyramid pooling module of PSPNet effectively processes multi-scale information in pathological images, its reliance on MobileNetV2 may affect its overall performance. LR-ASPP, while designed to be lightweight and incorporating spatial pyramid pooling, efficiently handles pathological images on low-resource devices while maintaining good accuracy. UNet, as a classic segmentation model, achieves a good balance between accuracy and recall through skip connections. Although HRNet is known for its high-resolution feature processing capabilities, its overall performance is relatively average. Meanwhile, FCN, as an early fully convolutional network, performs relatively well.

Our model is improved based on the SegFormer architecture, with the MIT-B0 lightweight hierarchical Transformer structure as the backbone, combining channel attention, channel shuffle, and spatial attention to further enhance the feature extraction capability of pathological images. Compared with SegFormer, our model performs better in capturing multi-scale features and global contextual information, excelling in complex tissue and edge segmentation tasks, achieving an MIoU of 92.19%, higher than SegFormer’s 91.07%. It is worth noting that all the numerical results in the table come from completely independent test sets, which were not used during the entire training and validation phases.

**Figure 3:**
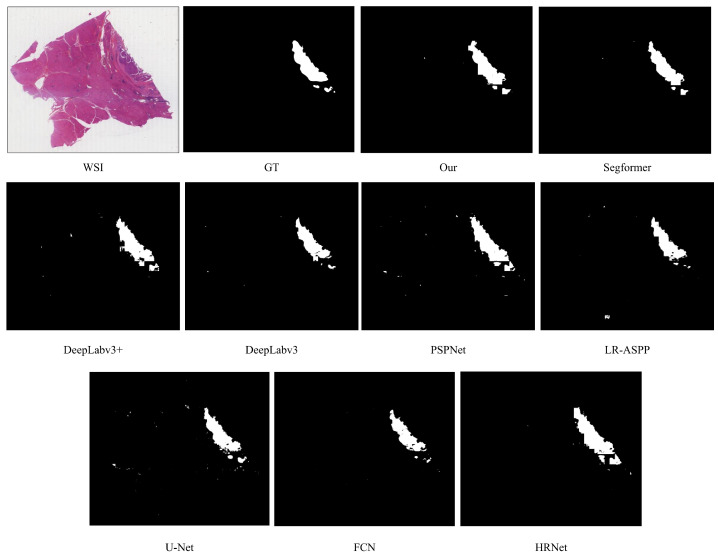
Visualization of prediction results on liver dataset. The initial image in the top row depicts the original image (resolution: 18,000 × 15,360 pixels). The second image illustrates the corresponding ground truth label, where the white region indicates a tumor, while the black region represents non-tumor areas, and the remaining nine images showcase the predictive outcomes from various models.

We selected a WSI from the test set with dimensions of 18,000 × 15,360 for visual representation of results. The predicted patches were stitched together to form a complete WSI. The first image in the top row displays the original image, and the second image shows the corresponding ground truth label. White areas represent tumor regions (foreground) with pixel values set to 255, while black areas represent non-tumor regions (background) with pixel values set to 0. The following nine images showcase the prediction results of various models (Figure 3).

In the prediction results of DeepLabV3+, DeepLabV3, PSPNet, LR-ASPP, and U-Net, varying degrees of false positive regions outside the tumors are observed. Although FCN performs relatively well in terms of false positive areas, it lacks completeness in predicting the tumor regions; conversely, HRNet predicts the tumor regions as overly large. Both SegFormer and our model exhibit some false positive areas outside the tumors, but the quantity is lower, and the predictions for tumor regions are relatively complete. Notably, all models display some degree of edge irregularities when predicting tumor boundaries.

#### 4.3.2. BACH Dataset

Our model achieves the best performance on the BACH dataset, attaining an MIoU of 71.71%, an MPA of 90.17%, and an accuracy of 93.52%, as shown in Table 2. In comparison, the SegFormer and HRNet models also demonstrate high performance, achieving MIoU values of 70.45% and 70.68%, respectively. Traditional models like UNet and PSPNet exhibit stable performance, though their MIoU and MPA are slightly lower. The FCN and DeepLabv3 models, which use ResNet-50 as the backbone network, perform poorly, showing lower MIoU and MPA values. All the values in the table are based on completely independent test sets, which were never involved in the entire training and validation process.

We selected a patch from the test set for visualization. The first image in the top row shows the original image, and the second image displays the corresponding ground truth label. In the label, black areas represent normal tissue and benign lesion regions (background) with pixel values set to 0. White areas represent in situ and invasive carcinoma regions (foreground) with pixel values set to 255. The remaining nine images showcase the prediction results of various models.

**Figure 4:**
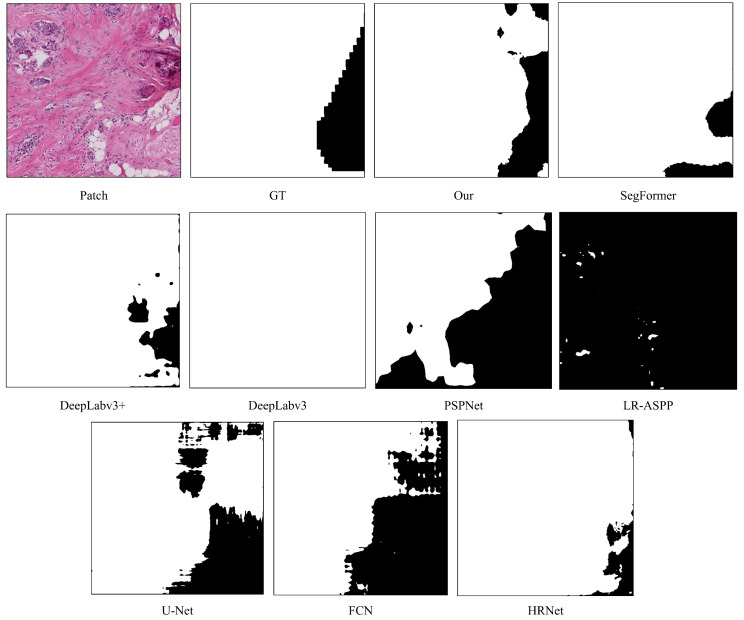
Visualization of prediction results on BACH dataset. The initial image in the top row depicts the original image (resolution: 512 × 512 pixels). The second image illustrates the corresponding ground truth label, where the white region represents in situ carcinoma and invasive carcinoma areas, and the black region represents normal tissue and benign lesion areas, and the remaining nine images showcase the predictive outcomes from various models.

As illustrated in Figure 4, the SegFormer, DeepLabv3+, DeepLabv3, and HRNet models exhibit over-segmentation in the foreground regions, while PSPNet, LR-ASPP, U-Net, and FCN demonstrate under-segmentation with relatively blurred edges. In contrast, our model performs better in the segmentation results but still shows some degree of under-segmentation.

## 5. Discussion

The GCSA-SegFormer model, employing a global channel spatial attention mechanism, enhances segmentation accuracy in tumor segmentation tasks on liver pathology images. Compared to traditional convolutional neural networks and baseline models, this model demonstrates superior performance in capturing global contextual information and fine structures, particularly showing some advantages in the segmentation of complex tumor tissues. While our results indicate performance gains over baseline models, we acknowledge that a direct comparison between GCSA and standard Transformer-based attention blocks has not been conducted in this study.

Despite making some progress, there are still some limitations. Firstly, the datasets used are still limited in terms of sample types and sizes, mainly focusing on HE-stained liver cancer pathology slices. Due to differences in staining methods and image acquisition standards across different hospitals, the model’s generalization capability still requires further verification. Additionally, when tumors are highly adherent to surrounding tissues or rare variant types are present, segmentation boundaries may exhibit deviations that need further optimization. Existing studies have shown that multimodal fusion can effectively integrate microstructural information with macroscopic structural features, demonstrating significant advantages in tumor heterogeneity assessment and classification [33,34]. Future research could explore the registration and fusion of pathology images with radiographic images such as PET-CT and MRI.

## 6. Conclusions

This study proposes the GCSA-SegFormer model, which performs well in tumor segmentation tasks on liver pathology images, particularly in capturing global contextual information and fine details at edges. This model leverages the Global Channel Spatial Attention mechanism to enhance the integration of multi-scale spatial features and global semantic information, resulting in more accurate identification of tumor boundaries and tiny lesions. Experimental results demonstrate that GCSA-SegFormer outperforms baseline models on liver cancer pathology image datasets and the BACH dataset, providing a solution for tumor segmentation tasks. In future work, we plan to implement k-fold cross-validation to further validate the robustness and generalizability of the proposed model.

## Figures and Tables

**Table 1:** Experimental results of different methods on the liver dataset.

Model	Backbone	MIoU (%)	MPA (%)	Accuracy (%)
DeepLabv3+	MobileNetV2	86.25	92.80	95.60
UNet	VGG-16	89.77	93.78	96.71
PSPNet	MobileNetV2	87.58	94.52	96.14
HRNet	HRNetV2	87.53	93.31	96.03
FCN	ResNet-50	89.00	93.35	96.60
DeepLabv3	ResNet-50	83.40	88.22	94.80
LR-ASPP	MobileNetV3	88.50	95.00	96.20
SegFormer	MIT-B0	91.07	94.60	97.26
Our Model	MIT-B0	**92.19**	**95.75**	**97.58**

**Table 2:** Experimental results of different methods on BACH dataset.

Model	Backbone	MIoU (%)	MPA (%)	Accuracy (%)
DeepLabv3+	MobileNetV2	68.57	84.39	92.28
UNet	VGG-16	68.74	86.99	92.63
PSPNet	MobileNetV2	68.66	85.09	92.39
HRNet	HRNetV2	70.68	87.02	93.02
FCN	ResNet-50	62.50	66.95	91.90
DeepLabv3	ResNet-50	60.30	64.80	91.40
LR-ASPP	MobileNetV3	68.30	72.50	93.10
SegFormer	MIT-B0	70.45	89.78	93.24
Our Model	MIT-B0	**71.71**	**90.17**	**93.52**

## Data Availability

The liver dataset, provided by the Eastern Hepatobiliary Hospital, is not readily available because it was obtained from the Pathology Department of the Eastern Hepatobiliary Hospital with permission. This dataset is part of an ongoing study on the identification of microvascular invasion. The BACH dataset can be accessed at https://www.kaggle.com/datasets (accessed on 10 November 2024). or by contacting the corresponding author. Additional code and data from our research can be obtained by reaching out to the corresponding author.
